# The utilization of atrial sensing dipole in single lead implantable cardioverter defibrillator for detection of new‐onset atrial high‐rate episodes or subclinical atrial fibrillation: A systematic review and meta‐analysis

**DOI:** 10.1002/joa3.12675

**Published:** 2022-01-15

**Authors:** Xuanming Pung, Daniel Zhihao Hong, Tzyy Yeou Ho, Xiayan Shen, Pei Ting Tan, Colin Yeo, Vern Hsen Tan

**Affiliations:** ^1^ Department of Cardiology Changi General Hospital Singapore City Singapore; ^2^ Yong Loo Lin School of Medicine National University of Singapore Singapore City Singapore; ^3^ Health Services Research Changi General Hospital Singapore City Singapore

**Keywords:** atrial high rate episodes, atrial sensing dipole, implantable cardiac defibrillator, subclinical atrial fibrillation

## Abstract

This meta‐analysis aims to evaluate the performance of atrial sensing dipole in single lead implantable cardioverter defibrillator (VDD‐ICD) recipients in particular diagnosing new‐onset atrial high‐rate episodes (AHREs) defined as rate threshold of 200 beats per minute, or subclinical atrial fibrillation (SCAF) defined as device‐detected AF without symptoms. We comprehensively searched PubMed, Embase, and ClinicalTrials.gov. Studies comparing contemporary single‐ and dual‐chamber ICD (VVI‐/DDD‐ICD) versus VDD‐ICD were included. Restricted maximum likelihood method for random effect model and Mantel‐Haenszel method for fixed effect model were used to estimate the effect size of new‐onset AHREs, or SCAF detection in each group. Three prospective studies were identified and total of 991 participants were included. There were 330 (33.3%) in VDD‐ICD and 661 (66.7%) in VVI‐/DDD‐ICD. Most (78%) participants were men. Median follow‐up was from 365 days to 847 days. VDD‐ICD has a higher likelihood of detecting AHREs or SCAF as compared to VVI‐/DDD‐ICD [(OR random effect : 2.6; 95% CI: 1.2, 5.8; *p* = .018); I‐squared = 67.8%, *p *= .019]. This difference was more apparently seen in the comparison between VDD‐ICD and VVI‐ICD [(OR random effect: 3.8; 95% CI: 2.1, 6.6, *p* < .001), I‐squared = 0.0%, *p *= .518]. The result is same as fixed effect. Rate of AHREs detection observed in VDD‐ICD was not statistically different when compared to the only group with DDD‐ICD from SENSE trial. In conclusion, this meta‐analysis reveals that the use of floating atrial sensing dipole in VDD‐ICD increases the detection of new‐onset AHREs or SCAF when compared to VVI‐ICD, with similar atrial sensing performance to DDD‐ICD.

## Graphical abstract

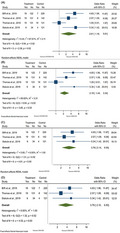

## 1 INTRODUCTION

Implantable cardioverter defibrillator (ICD) has become established as a guideline‐directed recommendation for primary and secondary prevention of sudden cardiac death.^1^ Data from international registries suggested that more patients received dual‐chamber ICD (DDD‐ICD) rather than single‐chamber ICD (VVI‐ICD) for primary prevention despite the indication for atrial pacing being absent.^2^

There is no clear consensus to guide the selection between single‐ and dual‐chamber ICD. Advantages in implantation of dual‐chamber ICD were presumed better discrimination between supraventricular and ventricular arrhythmias to reduce inappropriate therapies, allow monitoring of atrial fibrillation (AF), and to avoid an additional procedure to place an atrial lead if the need for bradycardia pacing arises. Ueda et al showed that only two patients (6.0%) of VVI‐ICD cohort required to undergo additional atrial lead insertion for bradycardia indication.^3^ Recent publication by Ahmed et al by looking at impact of insurance status on ICD implantation practice patterns based on National Cardiovascular Data Registry ICD registry found that among patients without a clear indication for pacing, the uninsured were more likely to receive single‐ versus dual‐chamber ICDs compared to those with insurance in which the apparent difference remains unclear, which requires further study.^4^

Atrial tachyarrhythmias detected on atrial leads, such as device‐detected AF, occur frequently in patients with cardiac implantable electronic devices (CIEDs) and are associated with an increased risk of stroke.^5^ This in turn influences decision relating to initiation of anticoagulation for stroke prophylaxis.

A single lead ICD (VDD‐ICD) system with a floating atrial sensing dipole [Linox^Smart^DX(Biotronik SE & Co, Berlin, Germany)] has been developed in early 2000s in order to improve the diagnostic capacity of atrial arrhythmias. The atrial dipole spacing covers a relatively large area of atrial surface of 49 mm^2^ which provides flexibility in positioning within the atrium in order to improve stability of atrial signal. There are two different configurations of the lead design: a 15 and 17 cm version based on the distance between the distal lead tip and mid‐point of atrial dipole. The VDD‐ICD was designed to amplify and filter the signals in order to maximize atrial sensing and minimize far‐field oversensing of ventricular signals. The concept of atrial sensing dipole has been proven safe and functional, meeting predefined clinical safety and efficacy of significantly higher than 90%.^6^

Hence, we performed this meta‐analysis to review the atrial sensing performance of VDD‐ICD using an atrial floating dipole in the detection of new‐onset atrial high‐rate episodes (AHREs) or subclinical atrial fibrillation (SCAF) and compare it with VVI‐ and/or DDD‐ICD.

## 2 METHODS

This meta‐analysis was performed in adherence to the Population, Intervention, Comparison, and Outcome (PICO) framework, PRISMA (Preferred Reporting Items for Systematic Reviews and Meta‐Analyses) guidelines^7^ and MOOSE (Meta‐analysis of Observational Studies in Epidemiology) checklist on the quality of reporting of meta‐analyses as shown in the Supplementary table S1 and S3.

Two independent reviewers (DZH and TYH) searched PubMed, EMBASE, clinicaltrials.gov, and Google Scholar for relevant articles on VDD‐ICD compare to VVI‐ and/or DDD‐ICD for the detection of new‐onset AHREs or SCAF. The following search terms were used in the literature review: (Atrial fibrillation) AND [(Implantable Cardioverter‐Defibrillator) OR (VDD‐ICD) OR (A+‐ICD) OR (floating atrial sensing dipole ICD lead)]. They also searched the reference lists of all published studies and biographies of review articles to identify additional articles. No language or publication status restriction were applied. The search was conducted from December 13, 2020 to March 28, 2021 and included studies up to March 28, 2021. Only studies that compared VDD‐ICD against VVI‐ and/or DDD‐ICD for detection of new‐onset AHREs or SCAF were included. The corresponding authors of the studies were also contacted to provide their unpublished data if any.

Study selection involved the screening of titles and abstracts, followed by full‐text evaluation of the eligible studies. The inclusion criteria were: (a) study population comprising two groups of patients receiving VDD‐ICD compared to VVI‐ and/or DDD‐ICD; and (b) reported new‐onset AHREs or SCAF.

The primary outcome was new‐onset AHREs defined as rate threshold of 200 beats per minute in these studies, or SCAF defined as device‐detected AF occurring without symptoms.

Data were independently extracted by two of the study investigators (DZH and TYH) using a standardized protocol and reporting form (Supplementary table S2). Any disagreements were resolved by arbitration, and consensus was achieved after panel discussion consisting of three reviewers (DZH, VHT, and TYH).

### 2.1 Statistical analysis

Data were pooled and analyzed using Stata version 16 statistical software. Effect size of new‐onset AHREs or SCAF detection was setup using two‐group comparison of binary outcomes. Restricted maximum likelihood method was used for random effect model and the Mantel‐Haenszel method for fixed effect model because of small number of events.

The overall effect size is presented using random effects as odds ratio (OR) with 95% confidence interval (CI). All statistical tests were two sided and used a significance level of *p* < .05. Heterogeneity between studies was evaluated using *I^2^* statistic. The *I^2^* statistic allows easy interpretation and overcomes the shortcomings of the Cochran's Q test.^8^ It is the proportion of total variation observed among the studies that is attributable to differences between studies rather than sampling error (chance), with *I^2^* values corresponding to the following levels of heterogeneity: low (<25%); moderate (25%–75%); and high (> 75%).^9^ A subgroup analysis comparing recipients of VVD‐ICD against VVI‐ICD was performed to assess the robustness of the result.

## 3 RESULTS

**FIGURE 1:**
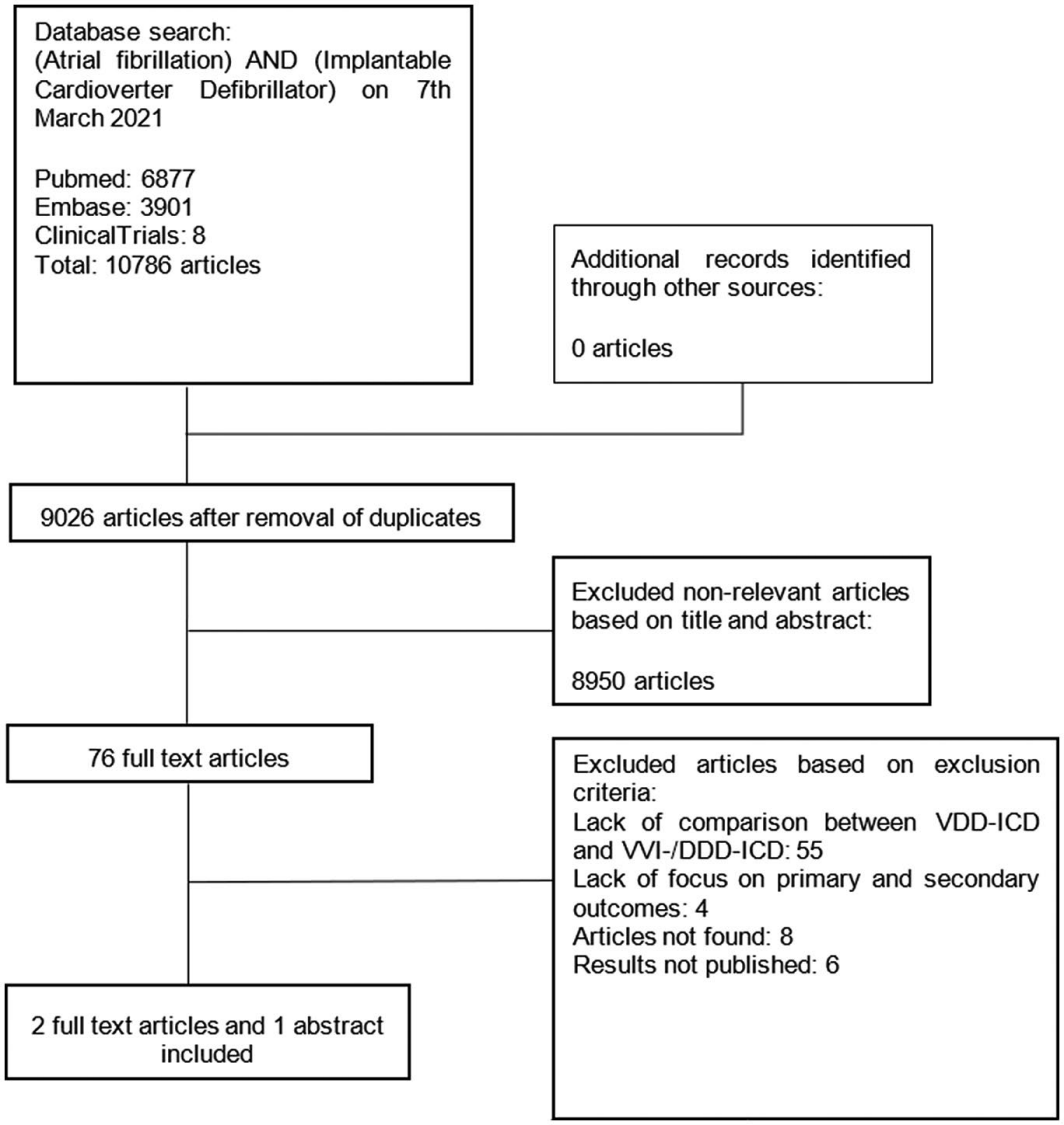
Flow diagram of selection. Progress through the systematic review.

Our database search resulted in 9026 unique articles. Of those, 76 were deemed relevant based on the title and abstract screenings. The full text of these 76 studies were assessed for eligibility, of which two full texts and one abstract met the final inclusion criteria and were included in the main analysis of this study as illustrated in Figure 1.

The three selected studies comprised cohort studies. There were no randomized controlled trials. Both the SENSE trial^10^ and the THINGS registry^11^ were prospective, cohort‐controlled, and multicenter studies comprising patients who met standard indications for a primary or secondary prevention ICD. The SENSE trial included two control groups (VVI‐/DDD‐ICD groups), while the VDD‐ICD system was only compared with conventional VVI‐ICD cohort in the THINGS registry and Statuto et al (abstract only).^12^

The characteristics of the patients in these three studies are shown in Table 1. A total of 991 participants were included in this meta‐analysis, of whom 330 (33.3%) were in VDD‐ICD system and 661 (66.7%) in VVI‐/DDD‐ICD group. Most (78%) participants were men. Median follow‐up was from 365 to 847 days. Patients had neither prior history of AHREs or AF, nor requirement for antibradycardia atrial pacing.

**TABLE 1:** Characteristics of patients included.

	Type of device: VDD‐ICD, *n* (%) VVI‐ICD, *n* (%) DDD‐ICD, *n* (%)	Male, *n* (%)	Age^b^, years	Follow up, days (median)	Left ventricle ejection fraction^b^, (%)	HTN, *n* (%)	DM, *n* (%)	CCF, *n* (%)	CKD, *n* (%)	CVA/TIA, *n* (%)	AC, *n* (%)	AP, *n* (%)	BB, *n* (%)	ACEI/ARB
THINGS registry Biffi M et al (*n* = 376)	140 (37.2%) 236 (62.8%) 0 (0%)	307 (81.6%)	Median: Total: 64.6 (53.8–72.7) VDD: 63.0 (54.1–69.2) VVI: 65.9 (52.5–74.1)	821	Median: All: 30 (25–35) VVI: 30 (25–35) VDD: 30 (25–35)	213 (57.4%)	101 (27.5%)	173 (46.8%)	47 (12.8%)	101 (27.5%)	53 (14.5%)	272 (74.5%)	333 (91.2%)	237 (64.9%)
SENSE trial Thomas G et al^a^ (*n* = 450)	150 (33.3%) 150 (33.3%) 150 (33.3%)	324 (72%)	Mean ± SD VDD: 59 ± 13 VVI: 54 ± 17 DDD: 59 ± 13	365	Mean ± SD: VDD: 33 ± 17 VVI:31 ± 16 DDD: 33 ± 16	318 (70.7%)	144 (32%)	353 (78.4%)	NA	33 (7.3%)	36 (8.0%)	262 (58.2%)	412 (91.6%)	258 (57.3%)
Statuto G et al (*n* = 165)	40 (24.2%) 125 (75.8%) 0 (0%)	(79%)	Median Total: 63 (48–72)	847	NA									

Note

AC, anti‐coagulation; ACEI, angiotensin‐converting enzyme inhibitor; AP, anti‐platelet; ARB, angiotensin receptor blockers; BB, beta blocker; CCF, congestive cardiac failure; CKD, chronic kidney disease; CVA, cerebrovascular accidents; DDD‐ICD, Dual‐chamber implantable cardiac defibrillator; DM, diabetes mellitus; HTN, hypertension; NA, not available; TIA, transient ischaemic attacks; VDD‐ICD, single lead floating atrial sensing dipole implantable cardiac defibrillator; VVI‐ICD, single‐chamber implantable cardiac defibrillator without atrial lead or floating atrial sensing dipole.

^a^ SENSE trial included data on DDD‐ICD patients.

^b^ Data presented as either mean, mean ± standard deviation or median (interquartile range).

**FIGURE 2:**
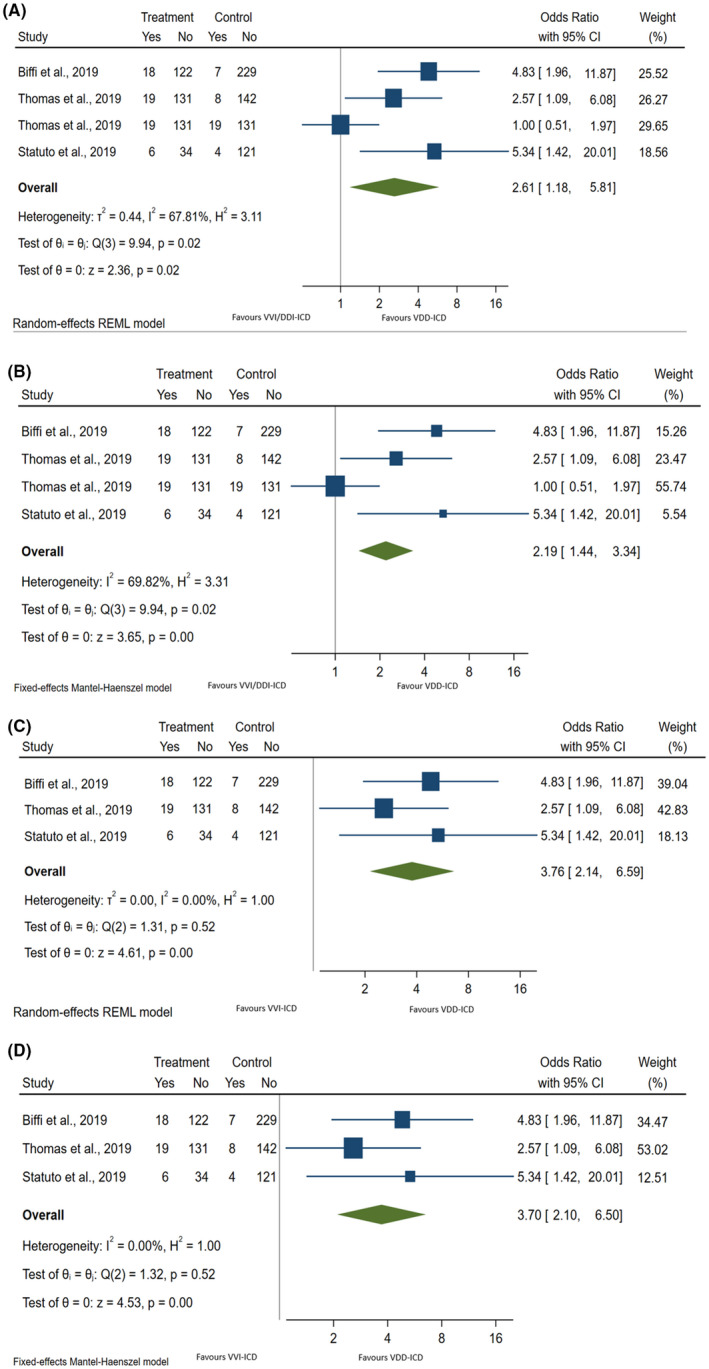
(A) Overall analysis: Forest plot for random effect of VDD‐ICD versus VVI‐/DDD‐ICD in detecting new‐onset AHRE or SCAF. (B) Overall analysis: Forest plot for fixed effect of VDD‐ICD versus VVI‐/DDD‐ICD in detecting new‐onset AHRE or SCAF. (C) Subgroup analysis: Forest plot for random effect of VDD‐ICD versus VVI‐ICD in detecting new‐onset AHRE or SCAF. (D) Subgroup analysis: Forest plot for fixed effect of VDD‐ICD versus VVI‐ICD in detecting new‐onset AHRE or SCAF.

Pooled result showed that VDD‐ICD system has higher likelihood in detecting AHREs or SCAF when compared to conventional VVI‐/DDD‐ICD as illustrated in Figure 2A and B. (OR random effect: 2.6 95% CI: 1.2, 5.8, *p =* .018; *I* ^2^ = 67.8%; P‐heterogeneity = 0.019).

Subgroup analysis demonstrated that VDD‐ICD has higher likelihood in detecting AHREs or SCAF when compared to VVI‐ICD alone. This is shown in Figure 2C. and D. (OR random effect: 3.8 95% CI: 2.1, 6.6, *p* < .001; *I* ^2^ = 0%; P‐heterogeneity = 0.519). As heterogeneity is not high, results of random effect and fixed effects are similar. Both results are presented in this meta‐analysis.

The P wave amplitude in VDD‐ICD system was assessed with regards to its atrial sensing performance. From the SENSE trial, a reduction in P wave amplitude to <2 mV was found in 7% of patients and P wave amplitude <1 mV in 3% of patients during 12 months of follow‐up.^10^ The THINGS registry showed a statistical but clinical negligible decrease in P wave amplitude from device implantation to 2‐year follow‐up; mean valve was 5.5 mV.^11^ Two VDD‐ICD recipients (1.4% of study population) from the registry had inadequate atrial sensing intermittently when P wave amplitude decreased to mean values of 0.4 mV, corresponding to the detection threshold programmed in the device (standard setting). Table 2 demonstrates the stability of atrial sensing performance of the VDD‐ICD system from our selected studies.

**TABLE 2:** VDD‐ICD atrial sensing performance (P wave amplitude, mV).

	At implantation	1 year	2 years
SENSE trial Thomas G et al (*n* = 150)	8.0 ± 5.0 (mean)	7.3 ± 4.8 (mean)	NA
THINGS registry Biffi M et al (*n* = 140)	5.5 (median), Interquartile range: 3.5‐10.1	NA	5.5 (median), Interquartile range: 2.9‐8.0

Note

NA, not available.

Incidence of inappropriate detection of AHREs was reported at 13% in the SENSE trial and most commonly because of electromagnetic interference (88.9%). No patient required device upgrade with the addition of an atrial lead for atrial pacing or poor atrial sensing. No inappropriate ICD therapies were observed in the VDD‐ICD group from the SENSE trial.^10^ Device‐related adverse events were also reported in this trial. The incidence of adverse events was similar between the VDD‐ICD and VVI‐ICD groups.

## 4 DISCUSSION

In this meta‐analysis, there was a statistically significant increase in the detection rate of AHREs, or SCAF in VDD‐ICD system as compared to VVI‐ICD alone. The AHREs detection rate was not significantly different between the VDD‐ICD and DDD‐ICD groups. There are several advantages of VDD‐ICD system including shortened procedural times and ability to detect AHREs or SCAF events leading to early diagnosis and management of AF.

The implantation of the VDD‐ICD system shortened the total procedural times because of less lead implantation time required.^13^ It also reduces the need for an additional atrial lead and associated lead complications, such as dislodgement. Early displacement occurs more frequently than late displacement, and these usually affect atrial leads.^14^ Atrial lead dislodgement was reported in 4% of DDD‐ICD recipients in the ADRIA study.^13^ Higher incidences of periprocedural complications, in‐hospital mortality, and need for earlier generator change in patients with DDD‐ICD compared to VVI‐ICD recipients were shown in studies.^2^, ^15^ Procedural complications in DDD‐ICD were associated with increased rates of procedural complications within 90 days of device implant (mechanical complication requiring reoperation for system, generator, and/or lead revision, device‐related infection, need for ICD replacement). There was no difference in 1‐year likelihood of re‐hospitalization for heart failure or all‐cause mortality.

Atrial tachyarrhythmias commonly occur in patients with CIEDs and include episodes of AHREs, or SCAF, which are associated with stroke.^5^ However, AHREs are defined differently by various studies in particular with regards to temporal cut‐off (duration and frequency of AHREs).^16^, ^17^, ^18^, ^19^ Based on a scientific statement from the American Heart Association, AHREs are defined as device‐detected atrial events, usually tachyarrhythmias, meeting programmed or other specified atrial high‐rate criteria (usually ranging between 175 and 220 bpm); SCAF refers to asymptomatic episodes of atrial fibrillation detected and confirmed by intracardiac electrograms and not previously detected by electrocardiographic or ambulatory monitoring.^20^

Device‐detected AHREs occurred in up to 20% of the patients by various studies and stroke risk differs between device‐detected (subclinical) and clinical AF for patients with identical stroke risk scores.^21^ Continuous atrial monitoring may identify patients with episodes of SCAF. However, these patients could have a different and potentially lower stroke risk compared to patients with clinical AF as reported in various stroke prevention trials.^16^, ^22^, ^23^

Benefits of oral anticoagulation in new‐onset AHREs, or SCAF is uncertain because of lack of clear evidence on treatment threshold.^24^, ^25^, ^26^ Data from the Veterans Health Administration showed wide variation in physician interpretation of device‐detected AF and 90‐day oral anticoagulation initiation across clinical practice. Highest risk of stroke was observed in patients with at least one episode of device‐detected AF >24 h, and the risk was reduced in those prescribed with anticoagulation.^27^ Ongoing trials such as ARTESIA (Apixaban for the Reduction of Thrombo‐Embolism in Patients With Device‐Detected Sub‐Clinical Atrial Fibrillation)^28^ and NOAH (Non‐Vitamin K Antagonist Oral Anticoagulants in Patients With Atrial High‐Rate Episodes)^19^ will provide evidence to guide decision on anticoagulation for these AHREs and SCAF episodes detected.

On the other hand, there may be several potential issues with VDD‐ICD system in terms of atrial sensing. Marai et al. stated concern with long‐term reliability on P wave amplitude during the follow‐up period of 12 months.^29^ It was a single center prospective experience that recruited all patients with P wave amplitude at implant of ≥0.8 mV. By 1‐year postimplantation, 11% of patients had a P wave amplitude <0.8, and one inappropriate shock was observed in a patient whose P wave amplitude dropped to 0.2 mV. Besides, the study also showed that the mean P wave amplitude was higher among the 15‐cm leads compared to the 17‐cm leads up to 12 months postimplantation. Similarly, the Linox DX study demonstrated that P wave in the 17 cm tip‐to‐ring configuration was significantly smaller than that observed in the 15 cm tip‐to‐ring distance and the rate of appropriate atrial sensing was also significantly lower.^6^

However, our meta‐analysis showed that there was a nonclinically significant trend in the decrease in P wave amplitude observed in the VDD‐ICD over the period of follow‐up although none of the studies comparing the two different leads configuration (17 vs. 15 cm). Other studies have shown acceptable P wave amplitudes in the VDD‐ICD system during follow‐up, although none specifically assessed the frequency of P wave amplitudes <0.8 mV.^6^, ^13^, ^29^ Worden et al. showed that satisfactory atrial sensing with floating atrial dipole is achievable and stable in time in the majority of patients.^30^ Safak et al. also reported long‐term stability in both atrial and ventricular signals with the newer generation VDD‐ICD over the entire follow‐up period of nearly 2 years.^31^ In term of P wave amplitude in comparing VDD‐ICD versus DDD‐ICD, based on SENSE trial, the amplified P wave amplitude at 1 year for VDD‐ICD remained stable (7.3 ± 4.8 mV) compared to during implantation (8.0 ± 5.0 mV).^10^ For DDD‐ICD group (separate atrial lead), the mean sensed atrial amplitude was 8.0 ± 5.0 mV at implant and remained stable at 12‐month follow‐up (7.3 ± 4.8 mV).

Currently, all CIEDs carry detection algorithms with high sensitivities and specificities for discriminating atrial and ventricular signals derived from CIEDs.^32^, ^33^, ^34^ Newer algorithmic approaches to AF detection have been developed. Multiple independent, real‐world analyses revealed an incidence rate of between 27% and 34% atrial fibrillation in the 6 months postimplant of single chamber Visia AF ICD.^35^, ^36^ In our meta‐analysis, there was a 13% newly detected AHREs in VDD group at 12 months from the SENSE trial,^10^ and a 2‐year incidence of AT/AF diagnosis of 11.4% in VDD group from THINGS registry.^11^ To date, no head‐to‐head comparison is available.

Increased frequency of inappropriate shocks has been associated with worse outcomes,^37^ impairs quality of life, and increases the mortality rate.^38^ Atrial sensing enables essential information from the atrium to be acquired and integrated into the detection algorithm to increase the accuracy in differentiating supraventricular arrhythmias from ventricular arrhythmias. Often, AF or SVT accounts for the leading cause of inappropriate shock therapy in ICD recipients,^39^ despite algorithms present to discriminate ventricular tachycardia (VT) from AF or SVT. The implantation of DDD‐ICD provides advantage over VVI‐ICD in its capability to analyze both atrial and ventricular rates and atrio‐ventricular relationship. However, DDD‐ICD may introduce new SVT‐VT discrimination problems.^40^ Evidence is conflicting as to whether dual‐chamber discrimination algorithms perform better than single chamber discriminators in reducing inappropriate shock therapy.^41^

The landmark ADRIA study evaluated the role of the SMART detection enhancement algorithm in VDD‐ICD with floating atrial sensing dipole, comparing its efficacy to DDD‐ICD.^13^ In that study, there was a higher prevalence of small atrial signals seen before amplification in the VDD‐ICDs, which can give rise to more episodes of under‐ or oversensing events. These episodes were predominantly P wave undersensing or far‐field R‐wave oversensing; they were mostly clinically silent. Studies reported the incidence of undersensing to be 5% ‐ 7%, the vast majority of which did not require any specific therapy.^42^, ^43^, ^44^ Electromagnetic interference (particularly in contact with the mains) remained the most common cause of atrial oversensing in the VDD‐ICD.^45^, ^46^ This was likely attributed to floating atrial dipole sensor that was amplified up to four times coupled with sampling rate as well as proprietary Biotronik digital noise gating algorithm.

**FIGURE 3:**
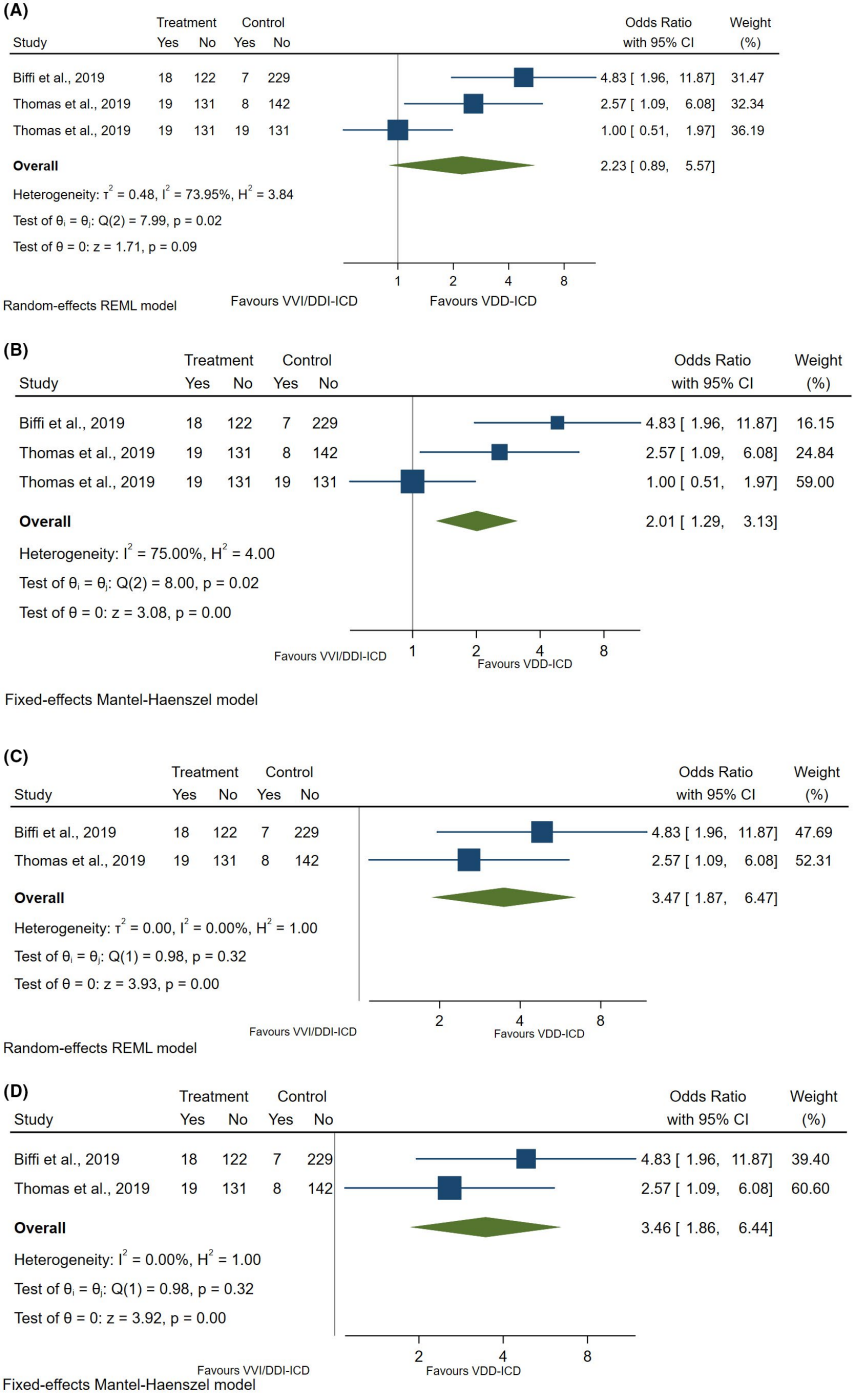
(A) Overall analysis without abstract: Forest plot for random effect of VDD‐ICD versus VVI‐/DDD‐ICD in detecting new‐onset AHRE or SCAF. (B) Overall analysis without abstract: Forest plot for fixed effect of VDD‐ICD versus VVI‐/DDD‐ICD in detecting new‐onset AHRE or SCAF. (c) Subgroup analysis without abstract: Forest plot for random effect of VDD‐ICD versus VVI‐ICD in detecting new‐onset AHRE or SCAF. (d) Subgroup analysis without abstract: Forest plot for fixed effect of VDD‐ICD versus VVI‐ICD in detecting new‐onset AHRE or SCAF.

Another important aspect is inclusion of conference abstract in systematic review and meta‐analysis. Conference abstracts without full publications remain a controversy subject whether to be included in systematic review and meta‐analysis. However, Cochrane and the United States National Academy Sciences both recommend always searching for and including conference abstracts in systematic review.^47^, ^48^ In addition, this issue was discussed in depth and published recently by Scherer et al.^49^ Essentially, Scherer et al concluded that it is worthwhile to search for and include results from conference abstracts in systematic reviews. Table 3 showed the resultant random effect for all studies when abstract was excluded which showed poor estimate (not significant) when the sample size was small as compared to fixed effect of all studies (Figure 3A—D). It became clearer that VDD‐ICD significantly increased detection of new‐onset atrial high‐rate episodes or subclinical atrial fibrillation (both random and fixed effect for all studies) when abstract was included.

**TABLE 3:** Random and fixed effect from study analysis with and without abstract.

	With abstract	Without abstract
All studies included		
Random effect	Significantly favour VDD‐ICD	Favour VDD‐ICD but not significant
Fixed effect	Significantly favour VDD‐ICD	Significantly favour VDD‐ICD
Single vs. DX^a^		
Random effect	Significantly favour VDD‐ICD	Significantly favour VDD‐ICD
Fixed effect	Significantly favour VDD‐ICD	Significantly favour VDD‐ICD

^a^ Single referred to single lead implantable cardiac defibrillator without atrial lead or floating atrial sensing dipole; DX (VDD‐ICD) referred to single lead floating atrial sensing dipole implantable cardiac defibrillator.

## 5 LIMITATIONS

The studies included in this meta‐analysis are nonrandomized studies, which have weaknesses that are inherent to observational data. There may be selection bias given the use of VDD‐, VVI‐, or DDD‐ICD in these nonrandomized, noncontrolled data sources. There is an on‐going randomized clinical trial comparing recipients of VDD‐ICD and VVI‐ICD on the rate of SCAF detection.^50^

Further studies could attempt to continue even longer term follow‐up of patients with the VDD‐ICD system to determine whether falling trend of atrial sensing over time from measurement of P wave amplitude translates into adverse clinical outcomes (e.g., undersensing and oversensing of atrial arrhythmias, inappropriate ICD shocks, or need for system revision).

Studies are also needed to establish the actual incidence of inappropriate AHREs or SCAF detection, and how we could mitigate these issues (e.g., electromagnetic interference, or lead dislodgement), which may lead to the incorrect prescription of anticoagulation therapy. With regards to cardiac resynchronization therapy (CRT), evidence is still limited in the utilization of a floating atrial sensing dipole as seen in the two‐lead CRT system.

## 6 CONCLUSION

In conclusion, this meta‐analysis reveals that the use of a floating atrial sensing dipole in VDD‐ICD increases the detection of new‐onset AHREs or SCAF when compared to VVI‐ICD alone, with similar atrial sensing performance to DDD‐ICD. However, longer follow‐up is needed to determine the reliability of the atrial sensing component in the VDD‐ICD.

## DISCLOSURE

None.

## Supporting information

Table S1‐S3
