# Supplementary material for: The utilization of atrial sensing dipole in single lead implantable cardioverter defibrillator for detection of new‐onset atrial high‐rate episodes or subclinical atrial fibrillation: A systematic review and meta‐analysis
Source: J Arrhythm. 2022 Jan 15;38(2):177–86. doi: 10.1002/joa3.12675 (PMC8977580; doi:10.1002/joa3.12675)
Supplement: Supplementary file 1 — Table S1‐S3 [file JOA3-38-177-s001.docx]

Supplementary Table 1. Population, Intervention, Comparison, and Outcomes (PICO)

|  | Inclusion criteria | Exclusion criteria |
| --- | --- | --- |
| Population | Patient characteristics   - First time implantable cardiac defibrillator with standard ICD indication - For primary prevention - Older than 18 years | Patient characteristics   - Prior pacemaker implantation - Existing or diagnosed atrial fibrillation or flutter - Contraindication for ICD implantation - Missing device type |
| Intervention | Implantable Cardiac Defibrillator | Pacemaker |
| Comparison | Papers that addressed the following comparisons were included   - Comparison of outcomes related to the use of VDD-ICD vs VVI-ICD - Comparison of outcomes related to use of VDD-ICD vs DDD-ICD |  |
| Outcomes | Papers that measured the following outcomes were included:  Primary outcome   - Detection of atrial high rate episodes and subclinical atrial fibrillation   Secondary outcome(s):   - Rate of inappropriate shock - Rate of perioperative complications - P-wave sensing |  |
| Study design | - Prospective studies - Retrospective studies - Randomised controlled trials - Case series - Articles in English or translated to English | - Reviews, grey literature, opinions, letters, commentaries and editorials |

Supplementary Table 2. Standardised Protocol and Reporting Form

| **STUDY SUMMARY** | | | | | |
| --- | --- | --- | --- | --- | --- |
| 1. | Lead Author | | 2. | Journal/ Year of Publication | |
| 3. | Study Type | - Case series - Case control - Observational - Cohort - Randomised Controlled Trial | 4. | Population | - VVI-ICD - DDD-ICD - VDD-ICD |
| **BASELINE DEMOGRAPHICS** | | | | | |
| 5. | Mean age (SD), years |  | 6. | Median age (IQR), years |  |
| 7 | Males, n (%) |  | 8. | Total population, n |  |
| 9. | Left Ventricular Ejection Fraction (%) |  |  |  |  |
| **COMORBIDITIES** | | | | | |
| 10. | Hypertension, n (%) |  | 11. | Diabetes Mellitus, n (%) |  |
| 12. | Congestive Cardiac Failure, n (%) |  | 13. | Chronic Kidney Disease, n (%) |  |
| 14. | Cerebrovascular Accident/ Transient Ischemic Attack, n (%) |  |  |  |  |
| **TYPE OF IMPLANTABLE CARDIOVERTER DEFIBRILLATOR (ICD)** | | | | | |
| 15. | Single lead ICD (VVI-ICD), n (%) |  | 16. | Double lead ICD (DDD-ICD), n (%) |  |
| 17. | Single lead ICD with atrial sensing dipole (VDD-ICD), n (%) |  |  |  |  |
| **OUTCOMES** | | | | | |
| 18. | Atrial High Rate Episodes/ Subclinical Atrial Fibrillation in VVI-ICD group, n (%) |  | 19. | Atrial High Rate Episodes/ Subclinical Atrial Fibrillation in DDD-ICD group, n (%) |  |
| 20. | Atrial High Rate Episodes/ Subclinical Atrial Fibrillation in VDD-ICD group, n (%) |  | 21. | P wave sensing amplitude of VDD-ICD at time of implantation |  |
| 22. | P wave sensing amplitude at follow up |  | 21. | Follow up period, days (median) |  |

Supplementary Table 3. MOOSE Checklist for Meta-analyses of Observational Studies

| **Item No** | **Recommendation** | **Reported on Page No** |
| --- | --- | --- |
| Reporting of background should include | | |
| 1 | Problem definition | 3-4 |
| 2 | Hypothesis statement | - |
| 3 | Description of study outcome(s) | 5-6 |
| 4 | Type of exposure or intervention used | 5-6 |
| 5 | Type of study designs used | 5-6 |
| 6 | Study population | 5-6 |
| Reporting of search strategy should include | | |
| 7 | Qualifications of searchers (e.g., librarians and investigators) | - |
| 8 | Search strategy, including time period included in the synthesis and key words | 5-6 |
| 9 | Effort to include all available studies, including contact with authors | 5-6 |
| 10 | Databases and registries searched | 5-6 |
| 11 | Search software used, name and version, including special features used (e.g., explosion) | 5-6 |
| 12 | Use of hand searching (e.g., reference lists of obtained articles) | 5-6 |
| 13 | List of citations located and those excluded, including justification | 7 |
| 14 | Method of addressing articles published in languages other than English | - |
| 15 | Method of handling abstracts and unpublished studies | 7 |
| 16 | Description of any contact with authors | - |
| Reporting of methods should include | | |
| 17 | Description of relevance or appropriateness of studies assembled for assessing the hypothesis to be tested | 5-6 |
| 18 | Rationale for the selection and coding of data (e.g., sound clinical principles or convenience) | 5-6 |
| 19 | Documentation of how data were classified and coded (e.g., multiple raters, blinding and interrater reliability) | 5-6 |
| 20 | Assessment of confounding (e.g., comparability of cases and controls in studies where appropriate) | 5-6 |
| 21 | Assessment of study quality, including blinding of quality assessors, stratification or regression on possible predictors of study results | 5-6 |
| 22 | Assessment of heterogeneity | 7-8 |
| 23 | Description of statistical methods (e.g., complete description of fixed or random effects models, justification of whether the chosen models account for predictors of study results, dose-response models, or cumulative meta-analysis) in sufficient detail to be replicated | 6-8 |
| 24 | Provision of appropriate tables and graphics | - |
| Reporting of results should include | | |
| 25 | Graphic summarizing individual study estimates and overall estimate | - |
| 26 | Table giving descriptive information for each study included | 7 |
| 27 | Results of sensitivity testing (e.g., subgroup analysis) | 7-8 |
| 28 | Indication of statistical uncertainty of findings | - |
| Reporting of discussion should include | | |
| 29 | Quantitative assessment of bias (e.g., publication bias) | - |
| 30 | Justification for exclusion (e.g., exclusion of non-English language citations) | - |
| 31 | Assessment of quality of included studies | - |
| Reporting of conclusions should include | | |
| 32 | Consideration of alternative explanations for observed results | - |
| 33 | Generalization of the conclusions (i.e., appropriate for the data presented and within the domain of the literature review) | 14-15 |
| 34 | Guidelines for future research | 14-15 |
| 35 | Disclosure of funding source | 1 |
